# Plumbagin has an inhibitory effect on the growth of TSCC PDX model and it enhances the anticancer efficacy of cisplatin

**DOI:** 10.18632/aging.205175

**Published:** 2023-11-03

**Authors:** Yuqi Xin, Qingkun Jiang, Chenshu Liu, Jiaxuan Qiu

**Affiliations:** 1 Department of Stomatology, The First Affiliated Hospital of Nanchang University, Nanchang, Jiangxi 330006, China; 2 Medical College, Nanchang University, Nanchang, Jiangxi 330006, China

**Keywords:** tongue squamous cell carcinoma (TSCC), patient-derived xenograft (PDX), plumbagin, proliferation, prognosis

## Abstract

Background: Head and neck squamous cell carcinomas are the sixth most common malignant tumors worldwide. Tongue squamous cell carcinoma is a common malignant tumor of this type, and it is associated with poor prognosis, a high rate of recurrence and a low survival rate. Plumbagin is derived from Plumbago zeylanica L, several studies report that plumbagin could inhibit cell, tumor metastasis, induce apoptosis in various cancer cells. Patient-derived xenograft (PDX) model can maintain the heterogeneity and microenvironment of human tumors, is a powerful research tool for developing potentially effective therapies for TSCC.

Methods: Tumor tissues obtained from TSCC patients were implanted into immunodeficient mice to establish TSCC PDX models. Subsequently, the PDX models were used to evaluate the anti-tumor effects of plumbagin on TSCC. Furthermore, we conducted next-generation sequencing (NGS) and explored the mRNA expression profiles between the treatment and control groups. We selected eight mRNAs related to the characteristics and prognosis of TSCC patients for further analysis.

Results: Plumbagin could inhibit the growth of TSCC PDX models and inhibit expression of Akt/mTOR pathway. In addition, plumbagin was shown to increase drug sensitivity to cisplatin. The eight mRNAs selected for further analysis, AXL, SCG5, VOPP1, DCBLD2 and DRAM1 are cancer-promoting genes, DUSP1, AQP5 and BLNK are cancer suppressor genes. And they were related to the diagnosis, growth, prognosis, and immune cell infiltration in TSCC patients.

Conclusion: Plumbagin exhibits an inhibitory effect on the growth of the PDX model of TSCC. Moreover, plumbagin enhances the inhibitory effects of cisplatin.

## INTRODUCTION

Head and neck squamous cell carcinoma (HNSCC) are the sixth most common malignant tumors worldwide. [1]. Tongue squamous cell carcinoma (TSCC) accounts for 25–40% of oral cancers. It is one of the most aggressive types of HNSCC [2–4], associated with poor prognosis, a high risk of relapse, and a 5-year survival rate of less than 50% [5–8]. According to previous studies, about 40–60% of patients show local recurrence or lymph node metastasis within five years of treatment [9, 10]. Therefore, complete recovery is not achieved despite the widespread use of advanced diagnostic techniques and therapies, such as surgery, chemotherapy, and radiotherapy [11–13]. Thus, there is an urgent need for researchers to understand the genetic and molecular mechanisms behind the carcinogenesis, and develop clinically relevant biomarkers and new treatment methods. However, little progress has been made in developing effective therapies as there are no models that mimick the biological characteristics of TSCC. Therefore, researchers need to develop a high-fidelity model that can reflect the biological characteristics of TSCC.

Intratumoral heterogeneity and microenvironment are critical in cancer progression, evolution, and response to therapy [14, 15]. Patient-derived xenograft (PDX) models are transplanted tumor models formed by implanting tissue blocks, circulating tumor cells, and primary cells from tumor patients into immunodeficient mice [16, 17]. The PDX models can maintain the heterogeneity and microenvironment of tumors, thus mimic human cancers and effectively predict response to therapy [16, 17]. Moreover, drug sensitivity testing using the PDX model has approximately 90% clinical relevance [18], indicating that the PDX model is a powerful tool for personalized and precise cancer treatment.

Phytochemicals are naturally occurring compounds of plant origin, in recent years, many studies have proved that they can directly or indirectly target multiple signal pathways of cancer cells and have been widely used to treat a variety of cancers [19]. At present, there is evidence that the combination of many phytochemicals and traditional anticancer drugs can play a significant role in improving the efficacy of single therapy, resisting drug resistance, and reducing organ toxicity [20, 21]. Plumbagin (5-hydroxy-2-methyl-1,4-naphthoquinone) is a traditional Chinese medicine (TCM) and is a small molecular weight compound derived from the roots of Plumbago zeylanica L [22]. Recent evidence shows that plumbagin could inhibit cell proliferation, tumor metastasis by inducing apoptosis of various cancer cells [23, 24]. Further, previous studies show that plumbagin could inhibit cell growth, migration, and invasion by inducing apoptosis of tongue squamous cell carcinoma cells in humans [25–27].

This study aimed to evaluate the anti-tumor effects of plumbagin on TSCC using next-generation sequencing and other techniques. Therefore, we established 14 PDX models of human tongue squamous cell carcinoma, which were then treated with plumbagin.

## MATERIALS AND METHODS

### Patient and tissue samples

Tumor samples were collected from 18 patients with TSCC during surgical procedures between December 2017 to April 2019, and processed tumor tissue within 24 hours. The criteria for selecting patients are that pathological findings confirmed squamous cell carcinoma, the tumor is located in the tongue, no distant metastasis, and they have not been treated before. Written informed consent was obtained from the patients and the patients agreed to use their tissues in the research. We have successfully established 14 TSCC PDX models. (Table 1). These were provided by the Department of Oral and Maxillofacial Surgery, the First Affiliated Hospital of Nanchang University. The study was conducted with prior approval of The First Affiliated Hospital of Nanchang University Ethics Committee (Permit No. (2022) CDYFYYLK (11-002)).

**Table 1:** Characteristics of the patients.

	***n* = 14**
Age (years):	
≤65	8
>65	6
Sex:	
Male	11
Female	3
Tumor size (cm):	
≤2	0
>2, ≤4	2
>4	12
Differentiation:	
Well	3
Moderate/low	11
Cervical lymph node metastasis:	
Yes	7
No	7
Clinical Staging:	
II	1
III	7
IV	6

### Materials

The plumbagin (Lot. No. 1012D021) was purchased from Solarbio Science and Technology Co., Ltd., (Beijing, China). The cisplatin (H20040813) was purchased from Haosen Pharmaceutical Co., Ltd., (Jiangsu, China). The hematoxylin staining solution (ZLI-9610) was purchased from Zhongshan Jinqiao Biotechnology Co., Ltd., (Beijing, China). The Scott blue solution (G1865) and the eosin dye solution (G1100) were purchased from Solarbio Science and Technology Co., Ltd., (Beijing, China). 30 mg plumbagin was dissolved in 1 mL DMSO and added 39 mL 0.9% saline, protected from light, stored at −20°C.

Balb/c nude mice aged 6 weeks (Hangzhou Ziyuan Laboratory Animal Technology Co., Ltd., Zhejiang, China, License number: SCXK2019-0004) were used for PDX establishment and treatment research. They were reared in an SPF environment.

### Establishment of patient-derived xenografts (PDX) for TSCC

The fresh surgically resected TSCC tissues were cut into 2 mm × 2 mm × 2 mm (8 mm^3^) sections within 24 hours, and evident necrosis, liquefaction tissues were removed. Sections were inoculated subcutaneously into the scapular region of 8-weeks-old female BALB/c nude mice and were numbered as P0. When the tumor volume grew to more than 1,000 mm^3^, the tumor was resected and cut into 2 mm × 2 mm × 2 mm (8 mm^3^) sections, subcutaneously were inoculated into the scapular region of BALB/c nude mice, numbered P1, and the tumors were passaged to P4 by the same method and used for subsequent experimental research. The implantation procedure was carried out as previously described [28].

### Anti-cancer treatment

When the average tumor volume reached 100–200 mm^3^, the mice were randomized into different treatment cohorts (*n* = 5): control group (0.9% saline, 0.1 ml once a day, intraperitoneal injection), cisplatin group (5 mg/kg once a week, intraperitoneal injection) [29], plumbagin group (2 mg/kg once a day, intraperitoneal injection) [30], and plumbagin + cisplatin group (the drug dosage and administration were the same as before). The tumor volume and the bodyweight were recorded every three days. The animals were sacrificed after 21 days (mice were injected intraperitoneally with sodium pentobarbital 150 mg/kg and mice were anesthetized and then euthanized by cervical dislocation), when the tumor volume in the control group reached 1,000 mm^3^. The tumors were then excised, weighed, and cut into two halves. One-half of the tumor samples were fixed in formalin, while the other half was stored in liquid nitrogen. Moreover, the hearts, livers, spleens, lungs, and kidneys were fixed in formalin. Tumor volume was calculated using the formula: length × width^2^ × 0.5, the measuring tool is a vernier caliper.

### Hematoxylin-eosin (HE) staining

The tissue samples were rinsed with water, dehydrated using ethanol solutions (70%, 80%, and 90%), embedded in paraffin, and finally sectioned. The paraffin sections were baked in an oven, dewaxed, hydrated, and then placed in an aqueous solution of hematoxylin for 3 min. After that, hydrochloric acid ethanol solution was applied for 15 s for differentiation, followed by washing with water. Moreover, the sections were placed in Scott solution for 15 s for blueing, rinsed with water, stained with eosin for 3 min, rinsed with running water, dehydrated, and sealed.

### Immunohistochemistry (IHC) staining

The tissue slices were immersed in 0.01 M citrate buffer (pH 6.0, Wellbio, Shanghai, China). The buffer was heated to boiling and cooked for 20 minutes, then cooled to room temperature. After cooling, the tissue slices were washed with PBS (pH 7.2~7.6, Wellbio, Shanghai, China). The diluted primary antibody (Ki67, Abcam, Cambridge, United Kingdom) was then added to the tissue samples and incubated at 4^o^C overnight. After that, the tissue slices were washed with PBS and were added 50~100 ul of anti-rabbit-IgG antibody-HRP polymer (Thermo Fisher Scientific, Shanghai, China). Further, 50~100 ul of a working solution of chromogenic reagent DAB (diaminobenzidine, Zhongshan Jinqiao Biotechnology Co., Ltd., Beijing, China) was added. The tissues were counter-stained with hematoxylin and mounted with neutral gum (Sigma, USA). Nuclear Ki-67 stained brown. The Ki67 slides were analyzed using a microscope (Olympus, Tokyo, Japan, BX43). Six 400X images were analyzed for each slide. The positivity cell rate was calculated as the ratio of the number of positive cells to the number of total cells.

### Short tandem repeat (STR) analysis

STRs are mainly used in genetic linkage map analysis, family identification, identity authentication, and other fields. We used the TIANamp genomic DNA kit to extract an appropriate amount of DNA from patient tumor tissue and PDX model tumor tissue, 20 STR loci and gender identification loci were amplified by MicroreaderTM21 ID System, PCR product detection was performed by GenReader 7010 genetic analyzer, detection results were analyzed by GeneMapper Software 6 (Applied Biosystems, USA) and compared with ExPASy databases.

### Next-generation sequencing

Genomic data were extracted from the PDX models for tongue squamous cell carcinoma (*n* = 3 in each group, from PDX models No. 3, 6, and 12) obtained from the four treatment groups and were mass-cut using Trimmomatic software version 0.36. Further, the data were compared to the reference genome using HISAT2 software version 2.1.0. For statistics mapping information, in addition, RSeQC software version 2.6.1, Qualimap software version 2.2.1, BEDTools software version 2.26.0 were used for analysis based on the results. After that, ASprofier software version 1.0.4 and EricScript software version 0.55 were used for genetic analysis. Moreover, StringTie software version 1.3.3b and R package WGCNA version 1.51 were used to analyze gene expression. Finally, differences in gene expression were analyzed using the R package DESeq version 1.26.0. The threshold for significant differential expression was set at *p* value < 0.05 and fold-change >2 or fold-change <0.5.

### Reverse transcription-quantitative polymerase chain reaction (RT-qPCR)

Tumor tissue was lysed by Trizol reagent (Kangwei Century, CW0580S, CWBIO, China), chloroform and isopropanol were added, then 75% ethanol was added after centrifugation. After the total RNA was extracted, a reverse transcription kit (Guangzhou Ribo Biological Co., Ltd., Guangzhou, China) was used to synthesize cDNA. The mixture was prepared according to the instructions of Takara TB Green Premix Ex TaqIIRT-PCR Kit. The RT-qPCR analysis was performed on Step One Plus Real-Time PCR System. Each experiment was repeated 3 times. The 2^−ΔΔCT^ method was used to calculate the expression level of the related RNAs. Moreover, the related GAPDH mRNA expression was used as an endogenous control. The primers used are shown in Table 2.

**Table 2:** Sequence of primers.

**Primer name**	**Primer sequence**
DRAM1 F	TGTCGCCAATTTTCAGGAGTT
DRAM1 R	TCCGTATGTGGCATGTCGAG
BLNK F	AAGTCAAAGGCCCTCCAAGT
BLNK R	CGGAGTCCGAATGTTCATCT
AQP5 F	GCCCTCTTAATAGGCAACCAG
AQP5 R	GCATTGACGGCCAGGTTAC
DUSP1 F	GCCACCATCTGCCTTGCTTACC
DUSP1 R	ATGATGCTTCGCCTCTGCTTAC
DCBLD2 F	ATGTGGACACACTGTACTAGGC
DCBLD2 R	CTGTTGGGATAGGTCTGTGG
VOPP1 F	GATGAACCCTGTCGGGAAT
VOPP1 R	GGCCTTCACTACCTGTTCGTA
SCG5 F	GGGTCCTTTTGGCAACATCC
SCG5 R	CCCCTGATCCTCACTAAAGTCC
AXL F	GACCGGCCAAGTTTTACAGA
AXL R	ATAACCTCCACCCTCATCCA
GAPDH F	TGACTTCAACAGCGACACCCA
GAPDH R	CACCCTGTTGCTGTAGCCAAA

### Western blot (WB) analysis

The tissue protein of the PDX model was extracted with cell lysis buffer (Beyotime, Shanghai, China). Protein content was determined using a BCA Protein Assay Kit (Bio-Rad, USA). Then, the protein sample was heated to 100°C and incubated for 5 minutes, electrophoresis was performed on SDS-PAM gel and transferred to the polyvinylidene fluoride (PVDF) membrane. The membrane was sealed with 5% skimmed milk for 80 min and incubated with the primary antibody (anti-rabbit monoclonal antibody AKT, p-AKT, mTOR, p-mTOR, 1:1,000, CST, USA; anti-rabbit monoclonal antibody β-actin, AXL, SCG5, VOPP1, DCBLD2, DRAM1, DUSP1, AQP5 and BLNK 1:2,000, Proteintech, China) at 4°C overnight. The membrane was incubated with the goat anti-rabbit secondary antibody (1:5,000, Proteintech, China) for 1 h the next day. And membrane was soaked with luminescent liquid, placed in an ultra-high sensitivity chemiluminescence imaging system (Chemi DocTM XRS+, Bole Life Medical Products Co., Ltd., Shanghai, China) for image generation. Finally, images were analysed with ImageJ software.

### Receiver operating characteristic (ROC) curves

The performance discrimination and diagnostic accuracy for mRNA were evaluated by constructing ROC curves and estimating the area under the curve (AUC) of patient with oral squamous cell carcinoma (OSCC) versus controls. Clinical data of OSCC patients were obtained from the TCGA Database (https://portal.gdc.cancer.gov/). Analysis was performed using the pROC package (version 1.17.0.1) in R (version 4.1.2).

### Immune infiltration analysis using ssGSEA

The immune signature score was calculated using the single-sample gene set enrichment analysis (ssGSEA2.0) implemented by the GSVA package (version 1.34.0) in R (version 4.1.2). Based on the characteristic genes of 24 different immune cell types, the relative immune cell tumor infiltration level was quantified from the gene expression profiles of each tumor sample. Spearman correlation analysis was used to analyze the correlation between AXL, SCG5, VOPP1, DCBLD2, DRAM1, DUSP1, AQP5, BLNK and the level of immune cell infiltration. Further, the Wilcoxon rank-sum test was used to analyze the correlation between immune cell infiltration and the expression of different mRNAs.

### Statistical analysis

All calculations were performed by SPSS 22.0 (IBM Corp., USA) and GraphPad Prism software version 7.0 (GraphPad Software, Inc., USA). Differences between groups were analyzed by using one-way ANOVA following the post-hoc tests by Tukey. Data were presented as mean ± standard deviation (SD). *P* < 0.05 was considered statistically significant.

### Availability of data and materials

The datasets used and/or analyzed during the current study are available from the corresponding author on reasonable request.

## RESULTS

### Establishment of patient-derived xenografts

**Figure 1:**
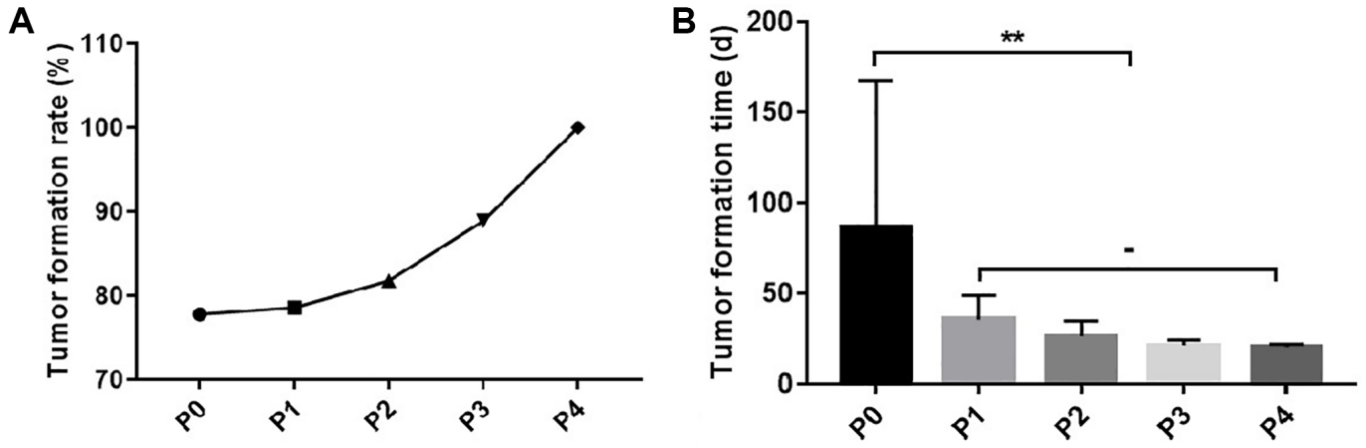
**Tumor formation rate and time of TSCC PDX model.** (**A**) The tumor formation rate of the P1-P4 generation PDX models. (**B**) The tumor formation time of the P1-P4 generation PDX models. ^**^p < 0.01; −, no statistical difference.

Eighteen TSCC samples were implanted under the skin of immunodeficient mice, to obtain the P0 generation. Subsequently, 14 PDX models of TSCC were successfully established. The tumor formation rate in the TSCC PDX models was about 77.8%, with an average tumor formation period of 86.4 days. The implanted tumors were passaged to four times to generate four generations (P1, P2, P3, and P4). The P4 generation model was used for subsequent experiments. The tumor formation rate increased as the passaging of the generations was increased. However, the tumor formation time decreased as the generations increased. On the other hand, the formation time of the models gradually stabilized after the P1 generation (Table 3, Figure 1).

**Table 3:** Tumor formation rate and time of TSCC PDX model.

	**Tumor formation rate (%)**	**Tumor formation time (d)**
P0	77.8	86.4 ± 70.9
P1	78.6	35.7 ± 10.3
P2	81.8	26.4 ± 4.9
P3	88.9	21.0 ± 1.9
P4	100	20.0 ± 1.1

### Histological analysis and STR analysis of the xenograft tumors

**Figure 2:**
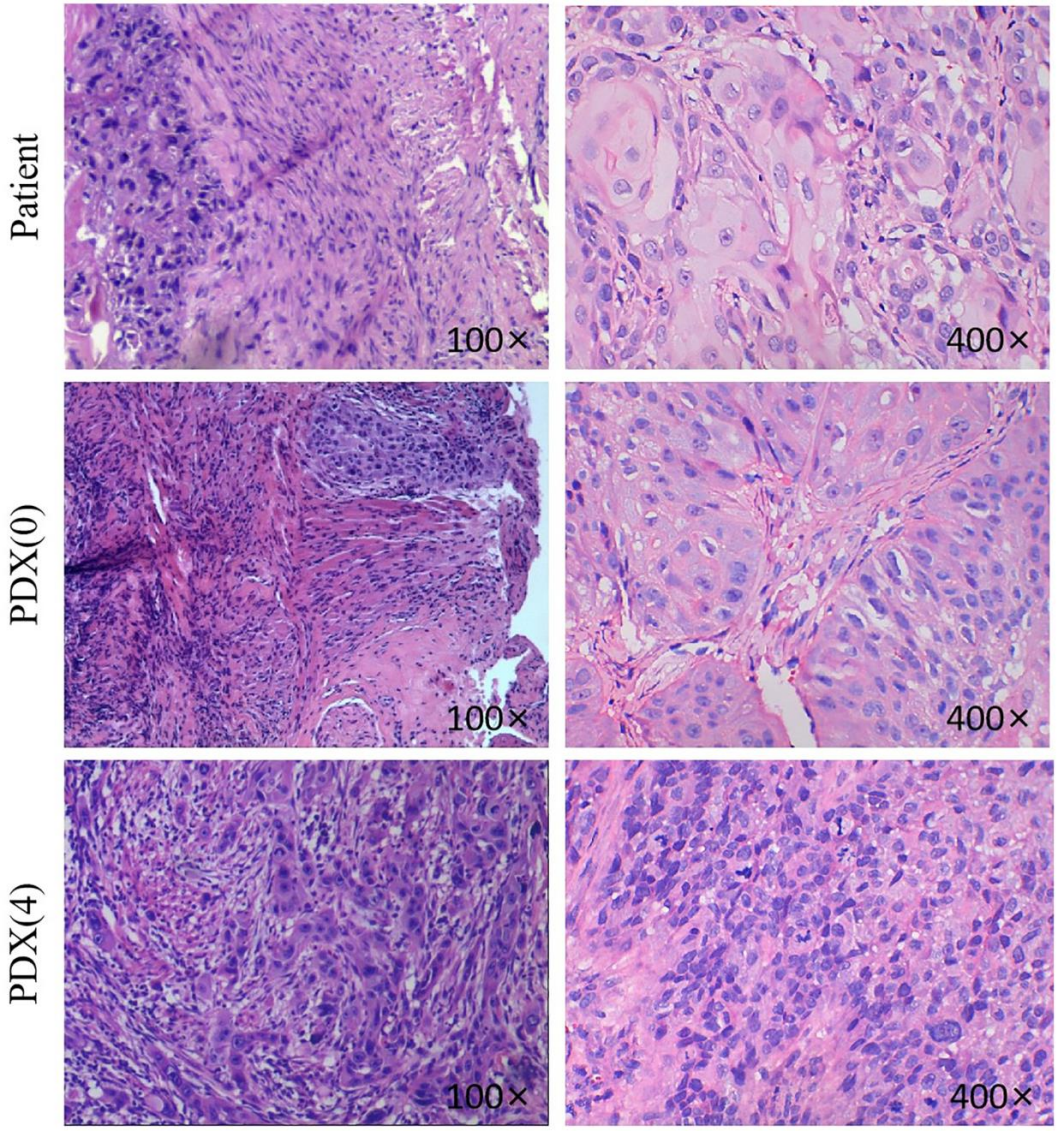
**H&E pictures of the TSCC patients and PDX models.** Histological analysis of tumor samples. After sacrificing the mice, TSCC tissues from patient and PDX models were fixed and checked with hematoxylin/eosin-staining. Cell nuclei were stained with hematoxylin (purple). The 400x and the 100x were not from the same section.

A total of 14 TSCC PDX models were established. An examination of the patient-derived xenografts and the original tumor samples showed that the proliferating epithelium of the PDX models invaded the connective tissue. In addition, the nucleus showed deep staining. However, the cytoplasm was less stained, and the cell boundary was unclear. There was a similar growth pattern and morphology between the PDX xenografts and the original tumor samples (Figure 2).

The DNA samples of patients and PDXs were tested for STR genotyping. The STR data showed no cross contamination of human cells was found in these tumor samples. STR analysis results showed that the two STR data conformed to the law of inheritance, had the same locus information and can be judged as the same individual source (Supplementary Figure 1).

### The anti-tumor effect of plumbagin on tongue squamous cell carcinoma

**Figure 3:**
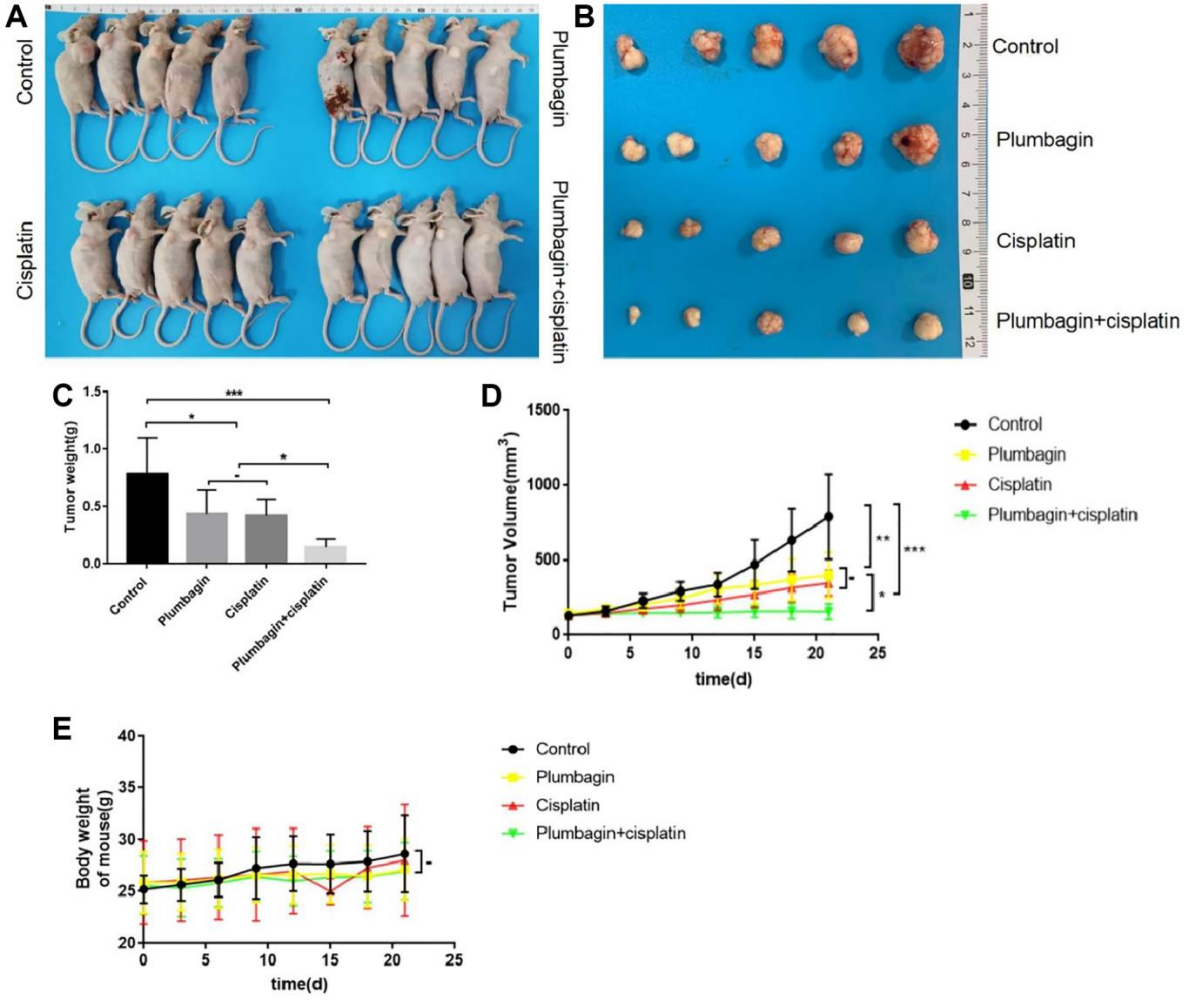
**The anti-tumor effect of plumbagin on tongue squamous cell carcinoma.** (**A**) TSCC PDX models were established. The mice were treated with plumbagin, cisplatin and their combination as described in Methods. (**B**) The tumor volume and the bodyweight were recorded every three days. The animals were sacrificed after 21 days of drug treatment. Representative samples of the PDX models showing the difference in tumor sizes between control, plumbagin, cisplatin and their combination. (**C**) Tumor weights of the PDX models were measured at the end of the experiment. (**D**) Tumor volumes of the PDX models after administration of plumbagin, cisplatin and their combination. (**E**) Body weights of the mice after administration of plumbagin, cisplatin and their combination. ^***^p < 0.001; ^**^p < 0.01; ^*^p < 0.05 and −, no statistical difference.

The anti-tumor activity of plumbagin was evaluated in the TSCC PDX models. Plumbagin was administered via intraperitoneal injection at a dose of 2 mg/kg daily, and cisplatin was administered via intraperitoneal injection at a dose of 5 mg/kg once a week. Treatment with plumbagin for 21 days showed an inhibitory effect on TSCC tumor growth. Moreover, the combination group showed the greatest inhibitory effect on tumor growth (Figure 3A–3D). These results suggested that plumbagin shows anti-tumor effects on PDX models of TSCC. Furthermore, plumbagin could enhance the anti-tumor effects of cisplatin.

**Figure 4:**
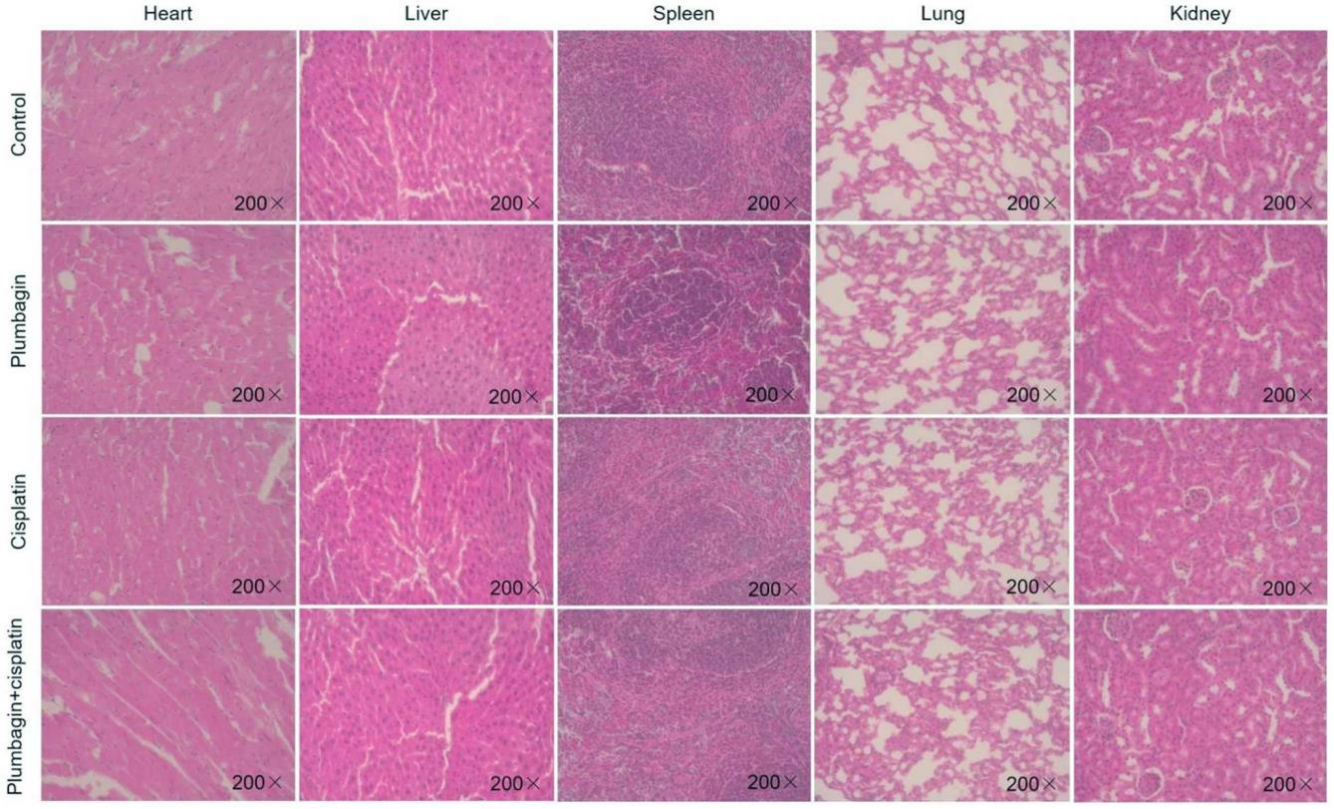
**The hearts, livers, spleens, lungs and kidneys of mice in each model group were sliced and stained with H&E.** Magnification × 200.

Analyzing the body weight of the mice of the PDX model, it was found that there was no statistical difference in the body weight changes of the mice in each group (Figure 3E). The H&E stained sections of the heart, liver, spleen, lung, and kidney of the mice were observed. The results showed that the H&E staining of the heart, liver, spleen, lung and kidney of each group of mice did not show any organ-related toxicity (Figure 4).

### The anti-proliferation effect of plumbagin

**Figure 5:**
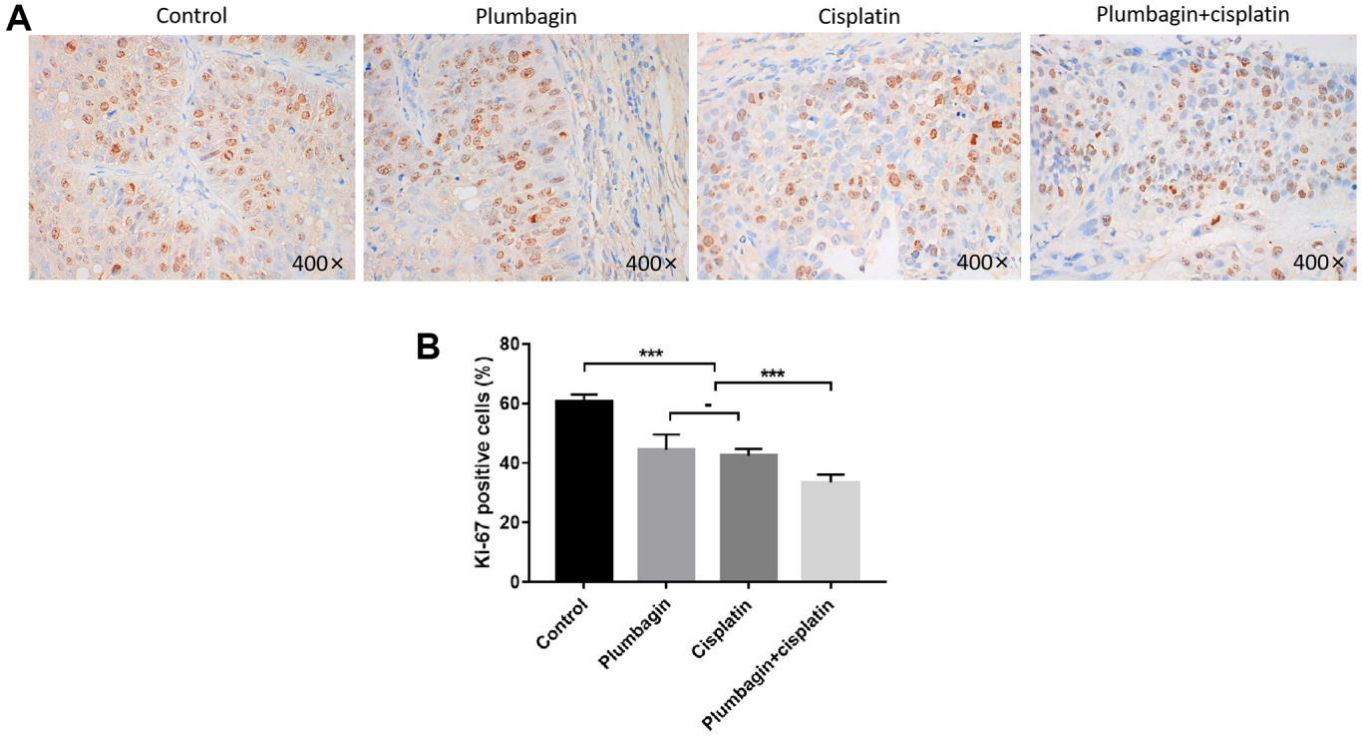
**Anti-proliferative effect of plumbagin on TSCC PDX model.** Ki67 IHC analysis was performed on each group of PDX models. (**A**) Use IHC to detect Ki67 expression. Nuclei of tumor cells were stained with hematoxylin (purple) and Ki67 positive cells have brown nuclei. (**B**) Quantitative analysis of Ki67 positive cells. Six 400X images were analyzed for each bar. Each bar represented the positivity cell rate, and the positivity cell rate was calculated as the ratio of the number of positive cells to the number of total cells. ^***^P < 0.001; −, no statistical difference.

The immunohistochemistry results showed a reduced rate of Ki67 positive cells in the treatment groups compared to the control group. Furthermore, the cisplatin plus plumbagin group showed the lowest detection rate of Ki67 positive cells (Figure 5). These results suggested that plumbagin could effectively inhibit the proliferation of tumor cells in TSCC PDX models. Moreover, plumbagin could enhance the anti-proliferative effects of cisplatin.

### Plumbagin affected Akt/mTOR expression in TSCC PDX models

**Figure 6:**
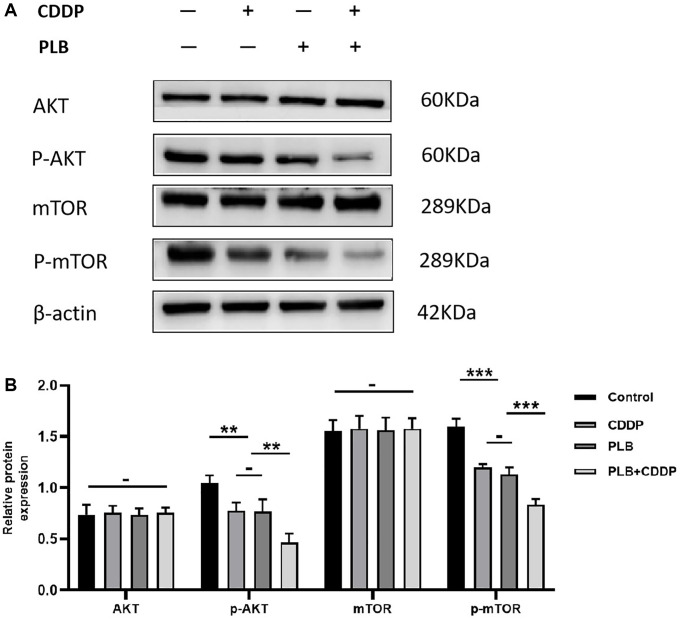
**Administration of plumbagin affected Akt/mTOR expression in TSCC PDX models.** (**A**) The expression level of AKT, p-AKT, mTOR and p-mTOR were measured by Western blotting. (**B**) The histograms indicate the relative expression levels of p-AKT/AKT, p-mTOR/mTOR. The quantitative data are shown as the mean ± SD of 3 independent experiments. ^***^P < 0.001, ^**^P < 0.01 and −, no statistical difference.

Our previous study demonstrated that plumbagin induced apoptosis in TSCC cells, and inhibited the AKT/mTOR signaling pathway. Further, plumbagin showed synergistic effects with cisplatin in inhibiting the growth of TSCC cells [23]. We examined the effect of plumbagin on Akt/mTOR activation in TSCC PDX models. In the presence of cisplatin or plumbagin, phosphorylated Akt/mTOR was downregulated. Moreover, the combination group showed phosphorylated Akt/mTOR was most significantly downregulated (Figure 6). These results suggested that plumbagin could inhibit the Akt/mTOR pathway. Moreover, plumbagin showed synergistic effects with cisplatin in inhibiting the growth of TSCC PDX models.

### The expression profile of messenger RNAs (mRNAs) in TSCC PDX models

**Figure 7:**
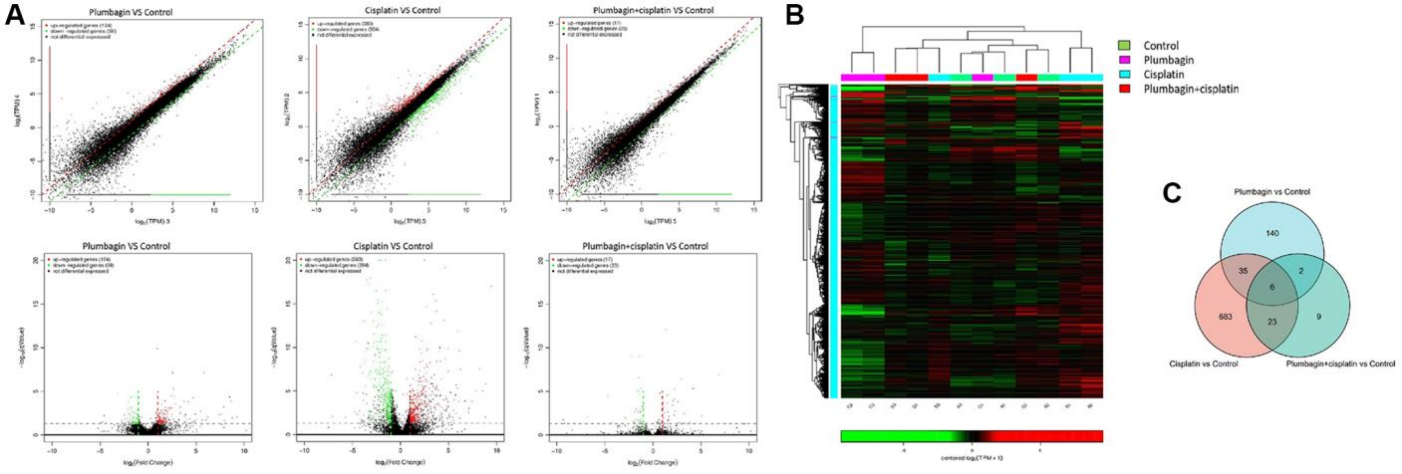
**The mRNA expression profile among the four groups of PDX models**. (**A**) Scatter plots and volcano plots showing the changes in mRNA expression between tumor tissues in the treatment groups and the control group. The horizontal and vertical axes on the scatter diagram are the two sets of sample log 2 (TPM) values. Each point in the figure represents a gene, and the closer the point is to the origin, the lower the expression level. Red represents the upregulated genes, green represents the downregulated genes, and black represents non-differentially expressed genes. The horizontal axis of the volcano map is the fold-change (log2(B/A)) value showing the differential gene expression between different groups of samples. On the other hand, the vertical axis shows the statistically significant *p*-value representing the change in gene expression. The smaller the *p*-value, the -log10. The larger the (*p*-value), the more significant the difference. (**B**) Heat map for hierarchical clustering of differential gene expression. In the figure, each row represents a gene, and each column represents a sample. The color represents the expression level of the gene. Red represents a high expression level, while green represents a low expression level. On the left is the dendrogram of gene clustering. The closer the two gene branches are, the closer their expression levels are. A dendrogram for sample clustering is shown at the top, the name of the sample is shown at the bottom, and the two sample branches are separated from each other. The closer the branches, the closer the expression of the genes in the two samples. (**C**) Venn diagram was the intersection of the three gene sets: the plumbagin group compared with the control group, the cisplatin group compared with the control group and the cisplatin plus plumbagin group compared with the control group.

**Figure 8:**
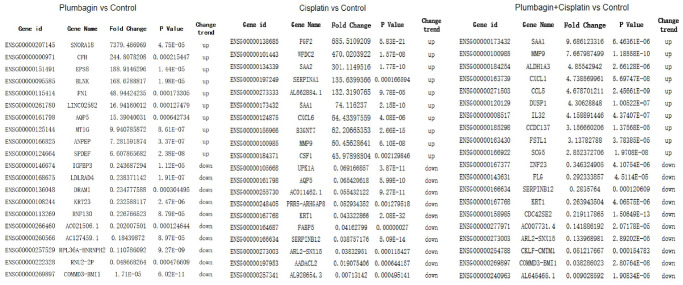
The top 10 upregulated mRNAs and top 10 downregulated mRNAs.

Next-generation sequencing and bioinformatics approaches were used to analyze and compare the mRNA expression profiles of tumor tissues in the four groups to determine the genes affected by plumbagin. Scatter and volcano plots were used to show the changes in mRNA expression in the four groups (Figure 7). A total of 124 upregulated mRNAs and 59 downregulated mRNAs were identified in the plumbagin group compared with the control group. Moreover, 383 upregulated mRNAs and 364 downregulated mRNAs were identified in the cisplatin group compared with the control group. In addition, 17 upregulated mRNAs and 23 downregulated mRNAs were identified in the cisplatin plus plumbagin group compared with the control group (Supplementary Tables 1–3). Moreover, hierarchical clustering analysis showed the differentially expressed mRNAs. The top 10 upregulated mRNAs and top 10 downregulated mRNAs were then listed (Figure 8). Further, five mRNAs were randomly selected to validate the results of RNA sequencing and evaluate their relative expression (Supplementary Figure 2). The RT-qPCR revealed that the results of RNA-seq were reliable and showed the abnormally expressed mRNAs in TSCC.

**Figure 9:**
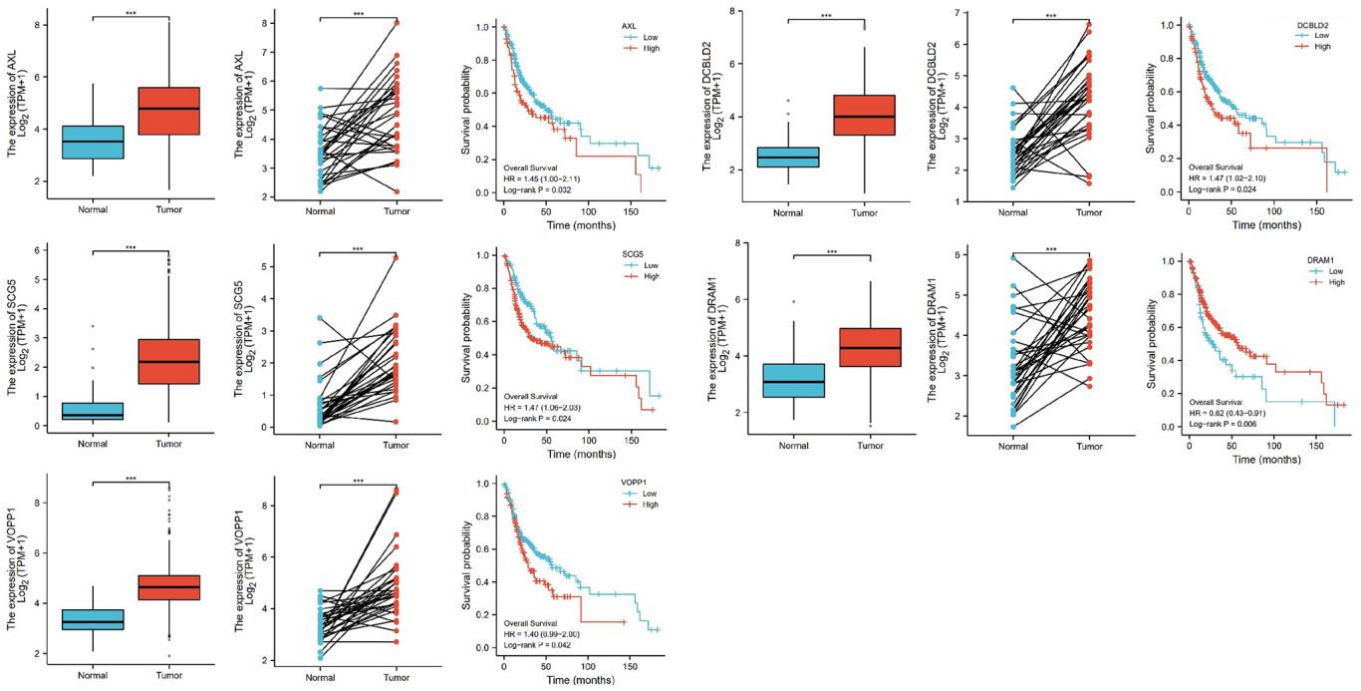
**The relative expression levels of 5 genes (AXL, SCG5, VOPP1, DCBLD2, DRAM1) in cancer tissues and normal tissues, and their influence on survival probability.**^ ***^P < 0.00.

**Figure 10:**
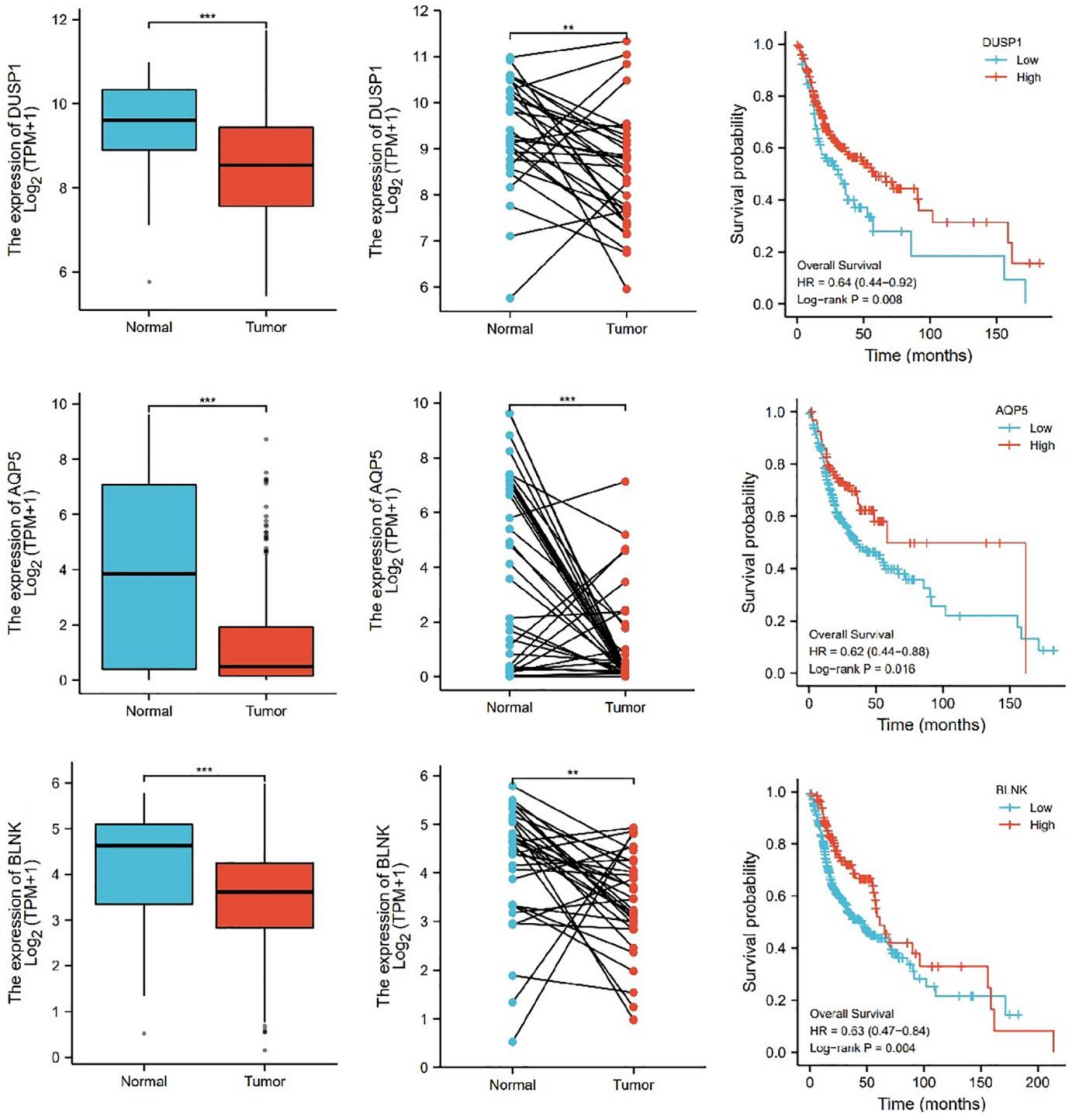
**The relative expression levels of 3 (DUSP1, AQP5, BLNK) genes in cancer tissues and normal tissues, and their influence on survival probability.**^ ***^P < 0.001.

**Figure 11:**
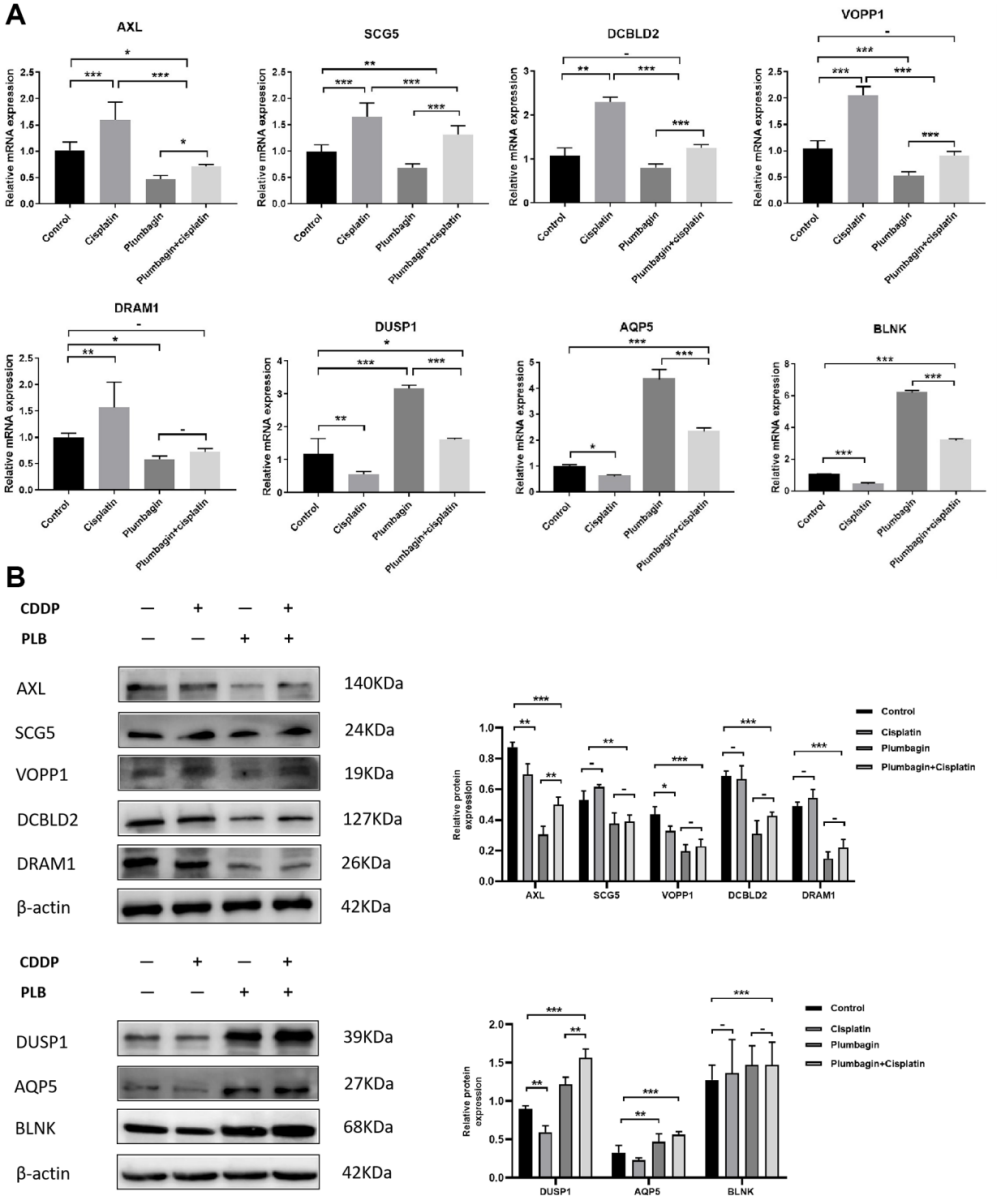
**Relative expression of AXL, SCG5, VOPP1, DCBLD2, DRAM1, DUSP1, AQP5, BLNK in the four treatment groups.** Treatment groups referred to PDX tumors harvested from mice. (**A**) The expression of differential genes in four groups of PDX models by RT-qPCR. (**B**) The expression of differential genes in four groups of PDX models by Western blotting. The quantitative data are shown as the mean ± SD of 3 independent experiments. ^***^P < 0.001; ^**^P < 0.01; ^*^P < 0.05; −, no statistical difference.

Further, we selected eight candidates with significant *p*-values (*P* < 0.001) from four groups of the abnormally expressed mRNAs to determine the mRNAs that promote or inhibit tumor growth in tongue squamous cell carcinoma. The results revealed five cancer-promoting genes (AXL, SCG5, VOPP1, DCBLD2, DRAM1), and three tumor suppressor genes (DUSP1, AQP5, BLNK). Further, based on OSCC project (RNAseq data in level 3 HTSeq-FPKM format, discard data without clinical information, and retain samples belonging to oral cancer sites in clinical information, exclude samples from non-oral cancer sites) of TCGA genome database (https://www.cancer.gov/about-nci/organization/ccg/research/structural-genomics/tcga), analysis was performed using the ggplot2 package (version 3.3.3) and survminer package (version 0.4.9) in R (version 4.1.2), the genes were related to the prognosis in patients with oral squamous cell carcinoma (Figures 9, 10). And we used RT-qPCR to evaluate their relative expression in each treatment. The RT-qPCR results revealed that cisplatin was shown not to inhibit the expression of cancer-promoting genes. However, treatment with both plumbagin and cisplatin proved that plumbagin could potentiate the inhibitory effect of cisplatin on cancer-promoting genes. And cisplatin did not promote the expression of the tumor suppressor genes. However, treatment with both plumbagin and cisplatin has shown that plumbagin could enhance the promoting effect of cisplatin on tumor suppressor genes (Figure 11A). And we used WB to detect differential gene expression at the protein level, the WB analysis results revealed that the differentially expressed genes at the protein level were consistent with those of RT-qPCR (Figure 11B).

### The receiver operating characteristic (ROC) curve for 8 biomarkers

**Figure 12:**
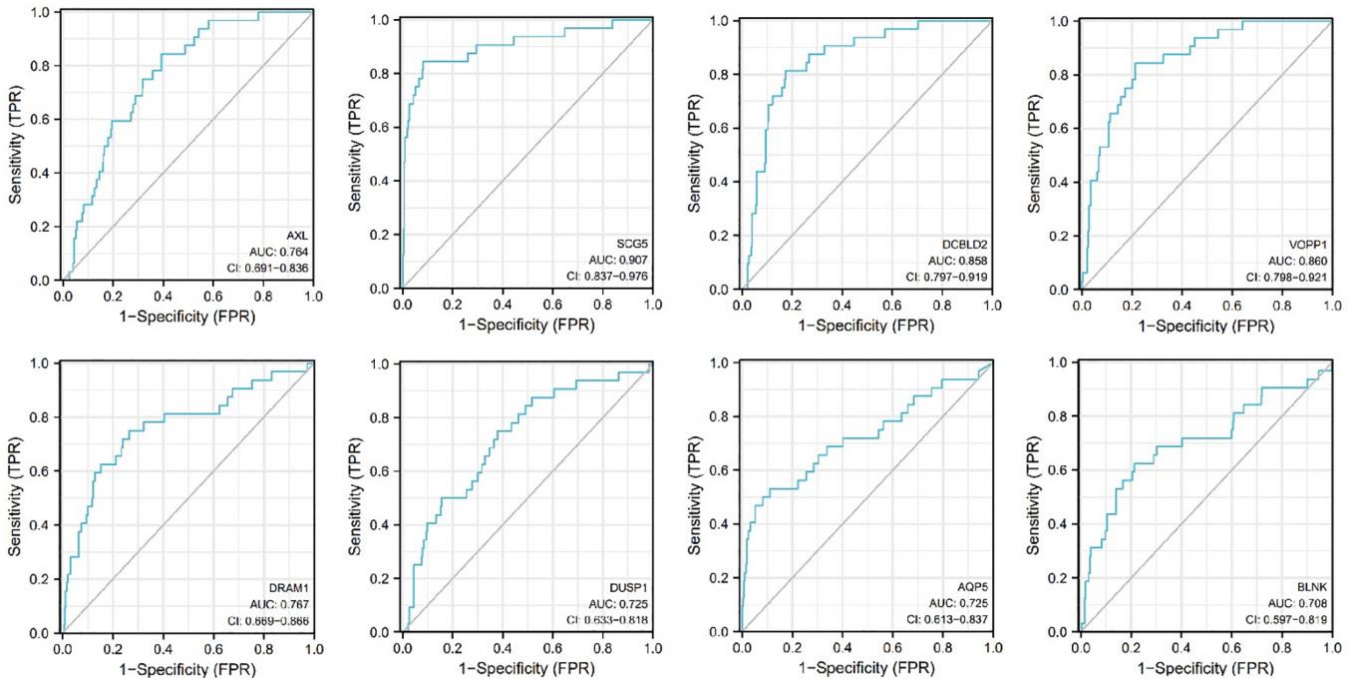
Receiver operating characteristic curves (ROC) for the AXL, SCG5, VOPP1, DCBLD2, DRAM1, DUSP1, AQP5, BLNK in the patients with oral squamous cell carcinoma.

The performance discrimination and diagnostic accuracy for mRNA were evaluated by ROC curves and AUC of patient with OSCC versus controls. The receiver operating characteristic (ROC) curve for 8 mRNAs are shown in Figure 12. The ROC area for AXL was 0.764, the ROC area for SCG5 was 0.907, the ROC area for DCBLD2 was 0.858, the ROC area for VOPP1 was 0.860, the ROC area for DRAM1 was 0.767, the ROC area for DUSP1 was 0.725, the ROC area for AQP5 was 0.725, the ROC area for BLNK was 0.708. The area values under the ROC curve of the 8 mRNAs are all between 0.5 and 1, and the AUC are all greater than 0.65, indicating that the accuracy of diagnosis using these genes is very high.

### Biomarkers correlated with immune infiltration level

**Figure 13:**
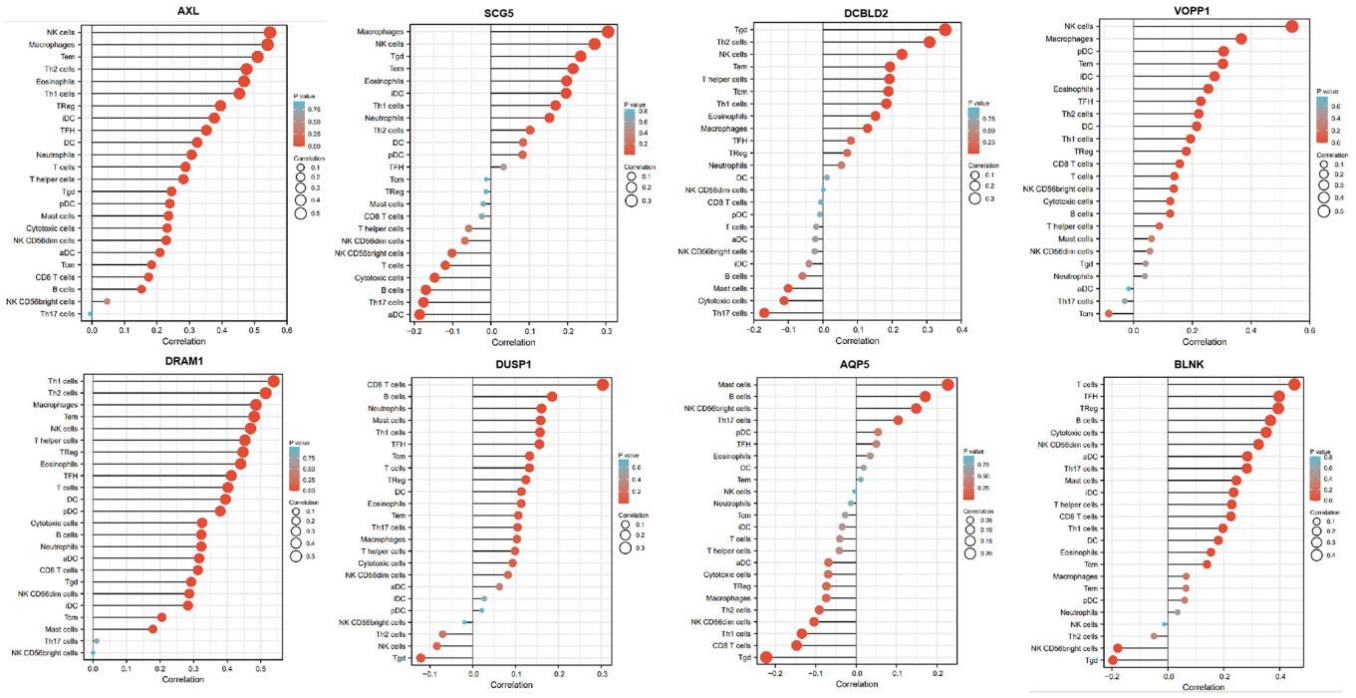
**Relationship between 8 mRNAs and immune infiltration.** The lollipop plot shows the correlation between AXL, SCG5, VOPP1, DCBLD2, DRAM1, DUSP1, AQP5 and BLNK expression and 24 immune cell subsets infiltration. The size of dots indicates the absolute Spearman *r* value.

The associations between the expression of the mRNAs and the abundance of immune cells infiltration were analyzed using ssGSEA. As illustrated in the lollipop plot (Figure 13), the expression of AXL, SCG5, VOPP1, DCBLD2, DRAM1, DUSP1, AQP5 and BLNK exhibited a strong positive correlation with some immunocytes, including macrophages, cytotoxic cells, T cells, B cells, Th1 cells, Th2 cells, and NK cells (*p*-value < 0.05). These findings suggest that these mRNAs affect immune responses by influencing the infiltration of immunocytes into the microenvironment of oral squamous cell carcinoma.

## DISCUSSION

Tongue squamous cell carcinoma is one of the most commonly diagnosed intraoral squamous cell carcinomas (25–40%). It is considered an aggressive form of squamous cell carcinoma with a five-year survival rate of less than 50% [31]. The tongue possesses a rich vascular, lymphatic network and a well-represented musculature. Therefore, tongue squamous cell carcinoma has an increased tendency for invasion and metastasis [32].

Compared to traditional preclinical experiments based on cell lines, the PDX models provide an important tool for tumor research, and the PDX models have emerged as pre-clinical models for cancer research. The PDX models maintain the heterogeneity and microenvironment of human tumors. Further, several studies have shown high consistency between pre-clinical experiments using PDX models and results of clinical treatment. Therefore, the PDX models are useful in studying tumor pathogenesis and developing personalized treatment [16, 17, 33]. The tumor model generated by transplanting tumor cell lines into mice is different from the primary tumor. Therefore, the drug dosage range tends to be different [33]. Therefore, the PDX model is a more reliable model for evaluating the anti-cancer effects of chemotherapeutic drugs [16, 17]. In addition, the PDX model plays a crucial role in investigating drug resistance in tumors [34].

Cisplatin is the most commonly used chemotherapy drug for TSCC. Moreover, it is used as the first-line agent for TSCC [35–37]. However, cisplatin is associated with toxic side effects, including peripheral neuropathy (CIPN), nephrotoxicity, ototoxicity, cardiotoxicity, and intestinal damage [38–41]. Moreover, with the passage of medication time and the increase of drug dose, drug resistance greatly limits the effectiveness of chemotherapy [42]. Oral squamous cell carcinoma shows an initial response rate to cisplatin of 80.6% [43]. However, more than 70% of the patients eventually relapse due to acquired drug resistance [44, 45]. Therefore, it is urgent to study a new treatment strategy to overcome the chemoresistance of TSCC and reduce the toxicity and side effects of cisplatin.

Plumbagin is a small molecular weight compound derived from the roots of Plumbago zeylanica L, a traditional Chinese medicine (TCM). Plumbagin has been reported to have anti-cancer effects in various cancer types [22]. Plumbagin played anticancer activity via many molecular mechanisms, such as apoptosis, autophagy pathway, antiangiogenesis pathway, anti-invasion [46]. Our previous study demonstrated that plumbagin induced apoptosis and autophagy by generating reactive oxygen species in TSCC cells, and inhibiting the AKT/mTOR signaling pathway. Further, plumbagin showed synergistic effects with cisplatin in inhibiting the growth of TSCC cells [27]. In this study, we continued to study the anticancer efficacy of plumbagin on the TSCC models.

To the best of our knowledge, this is the first study exploring the synergistic effects of cisplatin and plumbagin in TSCC-PDX models. The results demonstrated an inhibitory effect on tumor growth in plumbagin treatment group. However, the plumbagin plus cisplatin group showed synergistic effects with the greatest inhibitory effects on tumor growth. The body weight in the treatment groups remained unchanged compared to the control group. Furthermore, the H&E staining of the heart, liver, spleen, lungs, and kidneys in the four groups did not show any organ-related toxicity. Taken together, these results demonstrated that plumbagin has anti-tumor effects on TSCC PDX models. In addition, plumbagin enhances the anti-tumor effects of cisplatin and is not associated with severe toxicity. Ki67 is a related antigen of proliferating cells, which is mainly used to mark cells in the proliferation cycle. The higher the positive rate of Ki67, the faster the tumor growth, the poorer the tissue differentiation ability, and the higher the degree of malignancy [47]. The immunohistochemistry results revealed that plumbagin could effectively inhibit the proliferation of tumor cells in the TSCC PDX model. Furthermore, plumbagin combined with cisplatin could enhance the anti-proliferative effects of cisplatin. And we found that plumbagin could inhibit Akt/mTOR pathway in TSCC PDX models. Moreover, plumbagin showed synergistic effects with cisplatin in inhibiting Akt/mTOR pathway of TSCC PDX models. Therefore, this study is of great significance to the clinical application of plumbagin.

Next-generation sequencing (NGS) is a technology that allows thousands to billions of DNA fragments to be sequenced simultaneously. Next-generation sequencing is also known as high-throughput or massively parallel sequencing [48]. At present, several studies combine the PDX model with NGS to research disease diagnosis and treatment [49]. In this study, NGS was used to study the anticancer efficacy of plumbagin and the additive effects of plumbagin and cisplatin in TSCC-PDX models. In this study, we used NGS and bioinformatics approaches to analyze and compare the expression of mRNA in tumor tissues between the cisplatin, plumbagin and their combination treatment group with the control group. Overall, 124 upregulated mRNAs and 59 downregulated mRNAs were identified in the plumbagin group compared with the control group. In addition, 383 upregulated mRNAs and 364 downregulated mRNAs were identified in the cisplatin group compared with the control group. Furthermore, 17 upregulated mRNAs and 23 downregulated mRNAs were identified in the cisplatin plus plumbagin group compared with the control group. Eight genes were shown to be related to the prognosis in patients with tongue squamous cell carcinoma (AXL, SCG5, VOPP1, DCBLD2, DRAM, DUSP1, AQP5, BLNK).

Anexelekto (AXL) is a member of the Tyro3, AXL, and Mertk family of receptor tyrosine kinases (RTKs). It is associated with tumor cell growth, migration, invasion, and immune suppression [47, 50–52]. In addition, several studies reveal that secretogranin V (SCG5) is related to tumor prognosis [53, 54]. Moreover, Prosurvival Protein 1 (VOPP1) shows increased expression in various cancer types, such as squamous cell carcinoma, colorectal cancer, glioblastoma and gastric cancer [55]. VOPP1 enhances cell proliferation, migration and inhibits apoptosis [56, 57]. In addition, DCBLD2 is a neuropilin-related transmembrane protein expressed in endothelial cells (ECs). The DCBLD2 gene is involved in angiogenesis, tumorigenesis, tumor progression, and could be exploited as a therapeutic target for the regulation of angiogenesis [58, 59]. Moreover, DNA damage-regulated autophagy modulator 1 (DRAM1) plays an important role in autophagy and tumor progression [60]. Previous studies report that DRAM is a direct target of p53 and a critical factor for p53-dependent apoptosis and autophagy [61, 62]. The expression of AXL, SCG5, VOPP1, DCBLD2 and DRAM in oral squamous cell carcinoma were significantly higher in the TCGA database than in normal tissues. Moreover, these genes were related to the prognosis of patients. Furthermore, the RT-qPCR analysis and WB analysis revealed that cisplatin did not inhibit the expression of these genes. However, treatment using both plumbagin and cisplatin inhibited the expression of these genes.

Mitogen-activated protein kinase (MAPK) is essential for immune cell function. The activity of MAPK is controlled by dual-specificity phosphatases (DUSPs) [63]. Among them, DUSP1 has been shown to be related to cell proliferation, differentiation, transformation, stress response, inflammation, cycle arrest, and apoptosis [64, 65]. In addition, aquaporin 5 (AQP5), a member of the aquaporin family, is a cell membrane protein involved in the transport of water molecules [66]. Previous studies reported that AQP5 was associated with the formation and progression of various cancers [67, 68]. B-cell linker protein (BLNK) is an adaptor protein and it plays a crucial role in the B cell antigen receptor signaling pathway [69]. In fact, BLNK is considered an initiator of critical tumor suppressors. In addition, a previous study reported that BLNK could induce apoptosis [70]. The expression of DUSP1, AQP5, and BLNK in malignant tumors of the oral region was significantly lower than in normal tissues, according to the TCGA database. Furthermore, these genes were related to the prognosis of patients. The RT-qPCR analysis and WB analysis revealed that cisplatin did not promote the expression of these genes. However, a combination of plumbagin and cisplatin promoted the expression of these genes.

We used the ROC curve to detect the accuracy of the above 8 mRNAs in the diagnosis of oral squamous cell carcinoma. The areas under the ROC curve of AXL, SCG5, DCBLD2, VOPP1, DRAM1, DUSP1, AQP5, and BLNK in the detection of oral squamous cell carcinoma were 0.764, 0.907, 0.858, 0.860, 0.767, 0.725, 0.725 and 0.708, respectively. The area values under the ROC curve of the 8 mRNAs are all greater than 0.7. Therefore, these mRNAs were promising biomarkers in the screening and diagnosis of TSCC.

The mortality of patients with advanced or recurrent TSCC could increase drastically to 92% [71]. The introduction of immunotherapy (IT) recently provided a further treatment option for advanced and recurrent solid cancers, including TSCC [72]. Recent studies also mention the tumor microenvironment as a potential source for identifying new biomarkers [73]. Therefore, we analyzed the associations between the expression of the above mRNA and the infiltration of immune cells. We found the expression of these mRNA exhibited a strong positive correlation with some immunocytes, including macrophages, cytotoxic cells, T cells, B cells, Th1 cells, Th2 cells, and NK cells. Therefore, these mRNAs were shown to affect immune responses by influencing the infiltration of immunocytes into the microenvironment of oral squamous cell carcinoma. This study provides potential targets that could be exploited in future research to determine how plumbagin could be used as an immunotherapeutic agent in tongue squamous cell carcinoma, though the specific inhibitory mechanism needs to be further explored.

## CONCLUSION

In this study, 14 TSCC PDX models were successfully generated. Furthermore, plumbagin could inhibit the growth of TSCC PDX models and inhibited Akt/mTOR pathway. Moreover, plumbagin showed synergistic effects with cisplatin in inhibiting the growth of the PDX model of TSCC. Finally, the NGS demonstrated that plumbagin could enhance the effects of cisplatin by affecting genes such as AXL, SCG5, VOPP1, DCBLD2, DRAM1, DUSP1, AQP5, and BLNK which are related to the diagnosis, growth, prognosis, and immune cell infiltration of TSCC. Therefore, this study is of great significance to the clinical application of plumbagin.

## Supplementary Materials

Supplementary Figures

Supplementary Tables 1-2

Supplementary Table 3
