# Supplementary material for: Plumbagin has an inhibitory effect on the growth of TSCC PDX model and it enhances the anticancer efficacy of cisplatin
Source: Aging (Albany NY). 2023 Nov 3;15(21):12225–50. doi: 10.18632/aging.205175 (PMC10683608; doi:10.18632/aging.205175)
Supplement: Supplementary Figures [file aging-15-205175-s001.pdf]

# SUPPLEMENTARY FIGURES

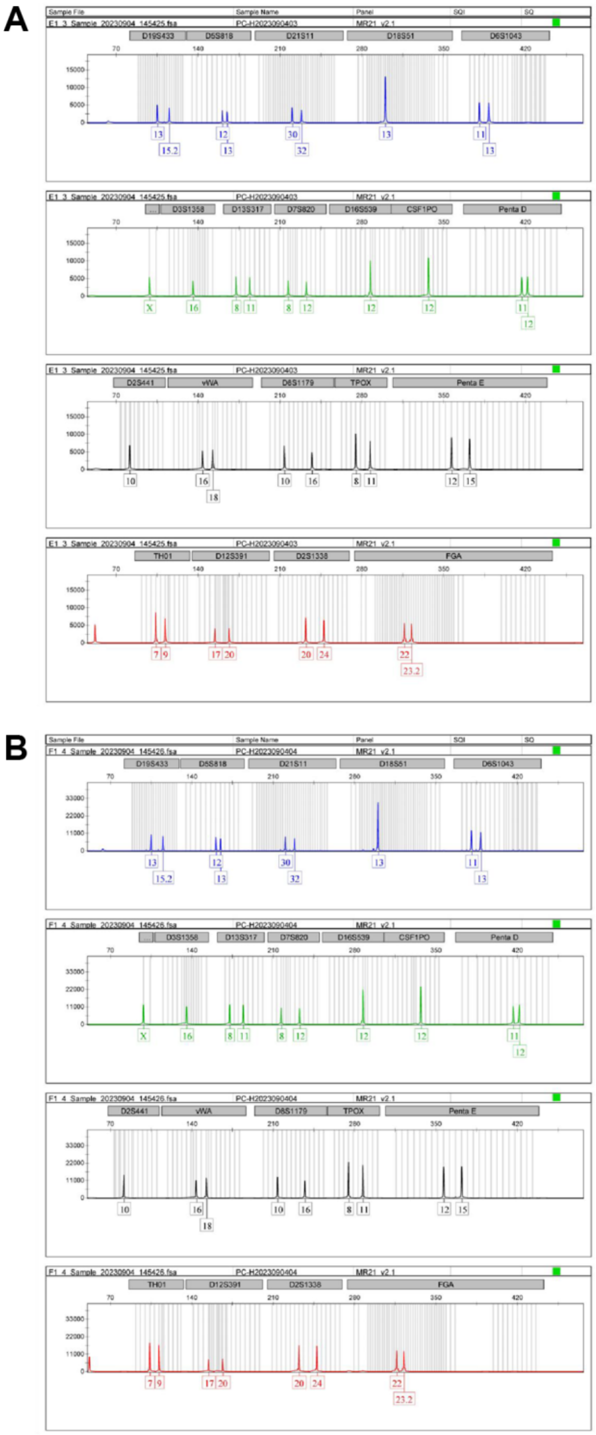


**Supplementary Figure 1. PDX tissue and tumor tissue are the same individual origin by STR profile.** (**A**) Short tandem repeat (STR) profile of patient tissue. (**B**) Short tandem repeat (STR) profile of PDX model.

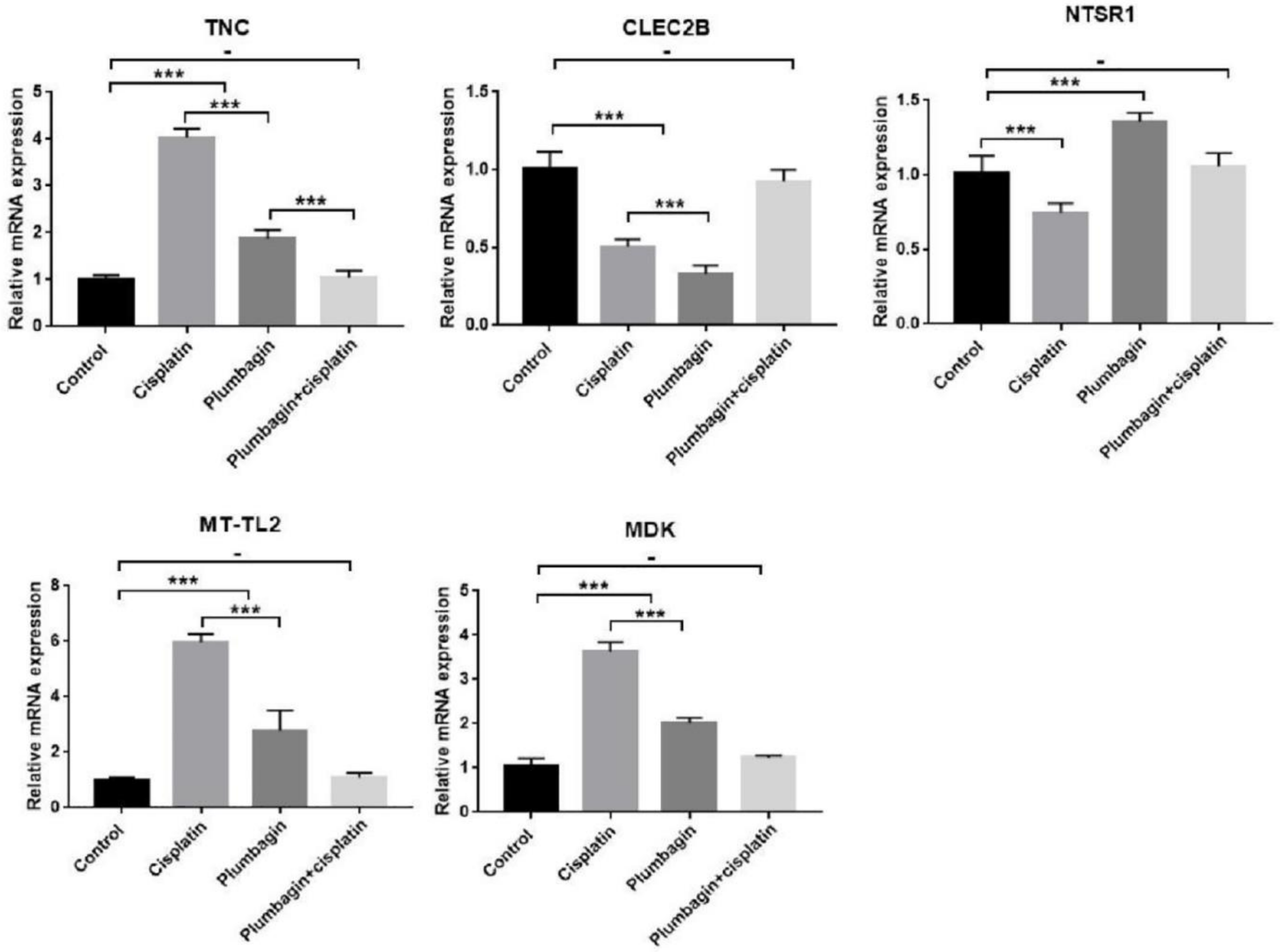


**Supplementary Figure 2. Relative expression of TNC, CLEC2B, NTSR1, MT-TL2 and MDK in the four treatment groups by RT-qPCR.** Treatment groups referred to PDX tumors harvested from mice. ***$P$ < 0.00; −, no statistical difference.
