# Supplementary material for: Plumbagin has an inhibitory effect on the growth of TSCC PDX model and it enhances the anticancer efficacy of cisplatin
Source: Aging (Albany NY). 2023 Nov 3;15(21):12225–50. doi: 10.18632/aging.205175 (PMC10683608; doi:10.18632/aging.205175)
Supplement: Supplementary Table 3 [file aging-15-205175-s003.pdf]

SUPPLEMENTARY TABLE

Supplementary Table 3. Expression of the differential genes in the plumbagin+cisplatin group compared with the control group.

Gene ID	Gene name	Fold change	log2 (Fold change)	*P* Value	Change trend
ENSG00000173432	SAA1	9.686123316	3.27591937	6.46E-06	up
ENSG00000100985	MMP9	7.667987499	2.938847985	1.19E-10	up
ENSG00000184254	ALDH1A3	4.85542942	2.279598895	2.66E-06	up
ENSG00000163739	CXCL1	4.738569961	2.244451738	5.70E-08	up
ENSG00000271503	CCL5	4.678701211	2.226108099	2.46E-09	up
ENSG00000120129	DUSP1	4.30628848	2.10644497	1.01E-07	up
ENSG00000008517	IL32	4.158891446	2.056199029	4.37E-07	up
ENSG00000185298	CCDC137	3.156660206	1.658398972	1.38E-05	up
ENSG00000163430	FSTL1	3.13782788	1.649766218	3.78E-05	up
ENSG00000166922	SCG5	2.852372706	1.512162504	1.9708E-08	up
ENSG00000161798	AQP5	2.804080912	1.487527979	2.2671E-07	up
ENSG00000095585	BLNK	2.766098807	1.467852692	5.45E-05	up
ENSG00000103034	NDRG4	2.752057809	1.460510775	0.00015106	up
ENSG00000255529	POLR2M	2.524976652	1.336270047	4.33E-17	up
ENSG00000167565	SERTAD3	2.119449764	1.083689772	2.48E-05	up
ENSG00000244462	RBM12	2.107639548	1.075628156	9.72E-07	up
ENSG00000006118	TMEM132A	2.017572951	1.012620839	4.40E-07	up
ENSG00000114439	BBX	0.496389635	−1.010455103	1.41E-05	down
ENSG00000114857	NKTR	0.472072373	−1.08292004	7.12E-07	down
ENSG00000073282	TP63	0.448430565	−1.15704348	0.00019702	down
ENSG00000163349	HIPK1	0.438690444	−1.188724815	2.91E-05	down
ENSG00000119938	PPP1R3C	0.428411092	−1.222932262	1.74E-09	down
ENSG00000175003	SLC22A1	0.407248439	−1.296018926	1.60E-06	down
ENSG00000170525	PFKFB3	0.39475456	−1.340972165	4.70E-05	down
ENSG00000147789	ZNF7	0.377585301	−1.40512549	1.46E-08	down
ENSG00000155508	CNOT8	0.370807387	−1.431258108	1.28E-13	down
ENSG00000118520	ARG1	0.360160124	−1.473289635	7.08E-11	down
ENSG00000134765	DSC1	0.352667077	−1.503621194	4.94E-05	down
ENSG00000127129	EDN2	0.350755349	−1.511462988	0.0001282	down
ENSG00000167601	AXL	0.346349511	−1.52969946	4.55E-09	down
ENSG00000167377	ZNF23	0.346324905	−1.529801958	4.11E-06	down
ENSG00000143631	FLG	0.292333857	−1.77431117	4.51E-05	down
ENSG00000166634	SERPINB12	0.2835764	−1.818190621	0.00012061	down
ENSG00000167768	KRT1	0.263943504	−1.921698934	4.07E-06	down
ENSG00000158985	CDC42SE2	0.219117865	−2.190220982	1.51E-13	down
ENSG00000277971	AC007731.4	0.141886192	−2.817193895	2.07E-05	down
ENSG00000273003	ARL2-SNX15	0.133968981	−2.900029096	2.89E-05	down

ENSG00000254788	CKLF-CMTM1	0.051217667	−4.287214651	0.00015478	down
ENSG00000269897	COMMD3-BMI1	0.038286023	−4.707038383	2.81E-08	down
ENSG00000240963	AL645465.1	0.009028592	−6.791283228	1.91E-05	down
